# Supplementary material for: Characterizing cell-type spatial relationships across length scales in spatially resolved omics data
Source: Nat Commun. 2025 Jan 3;16:350. doi: 10.1038/s41467-024-55700-1 (PMC11699133; doi:10.1038/s41467-024-55700-1)
Supplement: Supplementary file 3 — Supplementary Software 1 [file 41467_2024_55700_MOESM3_ESM.zip › CRAWDAD/plots/supplementary/S8_spleen_tissue_PKHL_clusters.pdf]

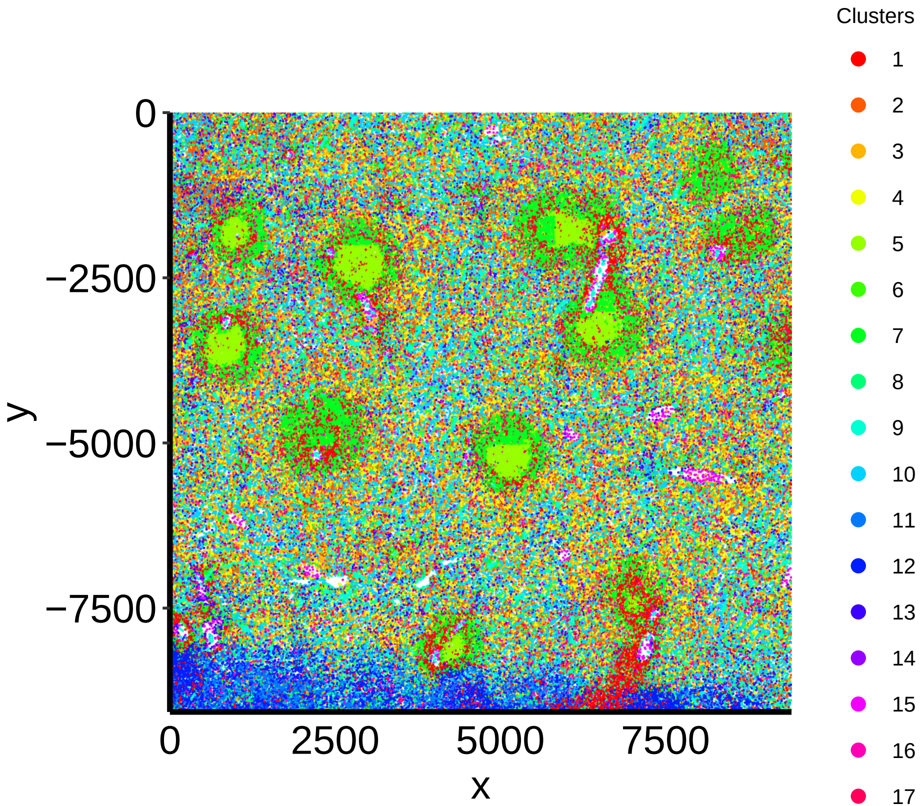
Clusters
1
2
3
4
5
6
7
8
9
10
11
12
13
14
15
16
17
0
−2500
−5000
−7500
y
0
2500
5000
7500
x
